# Efficacy of aficamten in patients with obstructive hypertrophic cardiomyopathy and mild symptoms: results from the SEQUOIA-HCM trial

**DOI:** 10.1093/eurheartj/ehaf364

**Published:** 2025-05-17

**Authors:** Martin S Maron, Juan Ramon Gimeno, Josef Veselka, Roberto Barriales-Villa, Brian L Claggett, Caroline J Coats, Sheila M Hegde, James L Januzzi, Ian J Kulac, Ahmad Masri, Michael E Nassif, John A Spertus, Daniel L Jacoby, Stephen B Heitner, Stuart Kupfer, Fady I Malik, Amy Wohltman, Iacopo Olivotto

**Affiliations:** Cardiovascular Division, Lahey Hospital and Medical Center, HCM Center at Lahey Clinic, 41 Mall Road, Burlington, MA 01805, USA; Cardiac Department, University Hospital Virgen Arrixaca, CIBERCV, ERN Guard-Heart, Murcia, Spain; Institute of Health Information and Statistics, Prague, Czech Republic; Complexo Hospitalario Universitario A Coruña, INIBIC, CIBERCV-ISCIII, A Coruña, Spain; Cardiovascular Division, Brigham and Women’s Hospital, Harvard Medical School, Boston, MA, USA; School of Cardiovascular and Metabolic Health, University of Glasgow, Glasgow, Scotland; Brigham and Women’s Hospital, Boston, MA, USA; Division of Cardiology, Department of Medicine, Massachusetts General Hospital, Harvard Medical School, Boston, MA, USA; Baim Institute for Clinical Research, Boston, MA, USA; Cardiovascular Division, Brigham and Women’s Hospital, Harvard Medical School, Boston, MA, USA; Cardiovascular Division, Oregon Health and Science University, Portland, OR, USA; University of Missouri Kansas City Healthcare Institute for Innovations in Quality and Saint Luke’s Mid America Heart Institute, Kansas City, MO, USA; Saint Luke's Mid America Heart Institute, Kansas City, MO, USA; Cytokinetics, Incorporated, South San Francisco, CA, USA; Cytokinetics, Incorporated, South San Francisco, CA, USA; Cytokinetics, Incorporated, South San Francisco, CA, USA; Cytokinetics, Incorporated, South San Francisco, CA, USA; Cytokinetics, Incorporated, South San Francisco, CA, USA; Meyer Children’s Hospital, Instituto di Ricovero e Cura a Carattere Scientifico (IRCCS), Florence, Italy

**Keywords:** aficamten, Clinical trial, Hypertrophic cardiomyopathy, Obstructive hypertrophic cardiomyopathy, Therapy

## Abstract

**Background and Aims:**

Patients with obstructive hypertrophic cardiomyopathy (oHCM) treated with aficamten in SEQUOIA-HCM (NCT05186818) demonstrated marked improvement in symptoms and functional capacity. This analysis explores whether oHCM and mild symptoms patients experience similar clinical benefits with aficamten as patients with more advanced limitations.

**Methods:**

Patients in SEQUOIA-HCM (*N* = 282) were grouped at baseline according to symptom severity. Mild symptoms (*n* = 118) were defined as New York Heart Association (NYHA) class II and Kansas City Cardiomyopathy Questionnaire Clinical Summary Score (KCCQ-CSS) ≥ 80, and moderate to severe symptoms (*n* = 150) as NYHA class II/III/IV and KCCQ-CSS <80. Primary endpoint was change in peak oxygen uptake (pVO_2_) from baseline to Week 24; secondary endpoints included change in NYHA class, KCCQ-CSS, outflow tract gradients, and N-terminal pro-B-type natriuretic peptide (NT-proBNP).

**Results:**

In aficamten-treated patients, change at Week 24 was not different between moderate to severe (1.8 mL/kg/min; *n* = 71) and mild (1.6 mL/kg/min; *n* = 62) symptom groups (*P* = .8). Likewise, the change in secondary endpoints (NYHA class, resting or Valsalva gradients, and NT-proBNP) did not differ significantly between the two symptom groups. Both groups experienced statistically significant improvements in KCCQ-CSS, but the extent of improvement was greater in the advanced symptom group (*P* = .02 for interaction). Treatment-emergent serious adverse events were infrequent in both groups.

**Conclusions:**

Patients with oHCM and mild symptoms treated with aficamten achieved significant improvement across a range of clinically relevant outcomes and generally similar to patients with more advanced symptoms. Less severely symptomatic patients could be considered for aficamten treatment.

## Teaser

In the recent phase 3 SEQUOIA-HCM trial (NCT05186818), patients with obstructive hypertrophic cardiomyopathy (oHCM) treated with novel cardiac myosin inhibitor aficamten, demonstrated marked symptom, function, and quality of life benefits. However, whether oHCM a mild symptom burden experience a similar benefit in health status compared with aficamten patients with more severe symptom limitation has not been described. In this substudy of SEQUOIA-HCM patients were grouped at baseline according to mild symptoms (NYHA class II and KCCQ-CSS ≥80) and moderate to severe symptoms (NYHA class II/III/IV and KCCQ-CSS <80). From baseline to week 24, pVO_2_ increased from 19.0 mL/kg/min to 20.7 mL/kg/min in aficamten-treated patients with mild symptoms (*P* = .003 vs. placebo) and from 17.8 mL/kg/min to 19.8 mL/kg/min in aficamten-treated patients with moderate to severe symptoms (*P* < .001 vs. placebo). The treatment effect of aficamten on pVO_2_ was similar between the two symptom groups [change (Δ) of 1.6 mL/kg/min (95% CI: 0.5 to 2.7) in mild symptoms group vs. Δ1.8 mL/kg/min (95% CI: 0.8 to 2.8) in moderate to severe symptoms group; *P* = .83 for interaction]. Likewise, the change in secondary endpoints (NYHA class, resting or Valsalva gradients, and NT-proBNP) did not differ significantly between patients on aficamten with mild vs. more severe symptoms. Both symptom groups experienced statistically significant improvements in KCCQ-CSS, but the extent of improvement was greater in the moderate to severe symptoms group (*P* = .02 for interaction). Patients with oHCM and mild symptoms treated with aficamten achieved significant improvement across a range of clinically relevant outcomes and this benefit was generally similar to patients with more advanced symptoms.

## Structured Graphical Abstract

**Structured Graphical Abstract:**
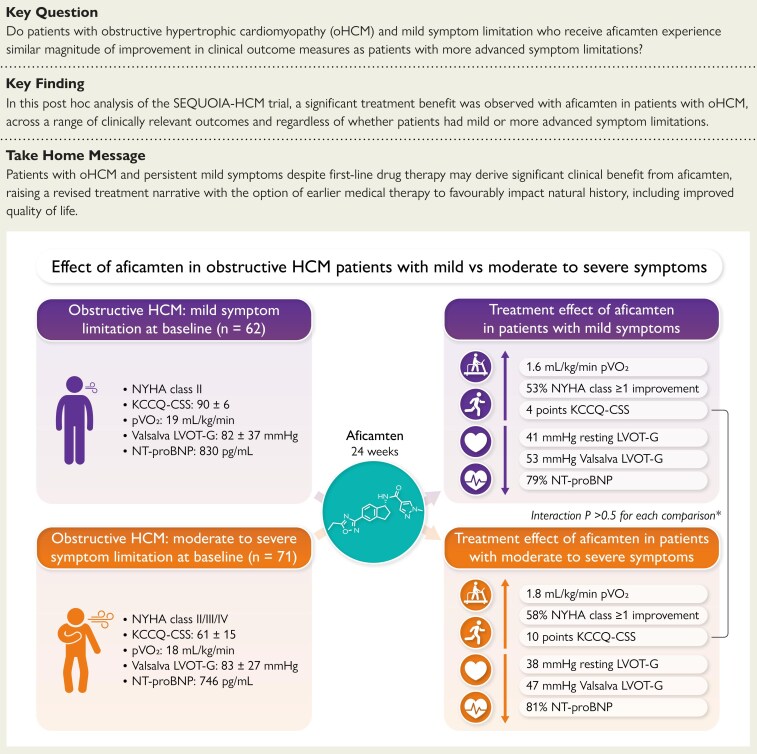
Aficamten in obstructive HCM with mild symptoms. Patients with oHCM and mild symptoms at baseline treated with aficamten over 24 weeks demonstrate significant improvement in clinical outcome measures and, compared with patients with oHCM and moderate to severe symptoms treated with aficamten, the magnitude of changes in clinical outcomes is similar. * Except *P* value for comparison of KCCQ-CSS between groups, which was statistically significant. KCCQ-CSS, Kansas City Heart Failure Questionnaire-Clinical Summary Score; NYHA, New York Heart Association; oHCM, obstructive hypertrophic cardiomyopathy.

**See the editorial comment for this article ‘Cardiac myosin inhibitors in hypertrophic cardiomyopathy: the need for precision medicine’, by A. Yilmaz, https://doi.org/10.1093/eurheartj/ehaf507.**

## Introduction

The principal objective of therapeutic interventions in obstructive hypertrophic cardiomyopathy (oHCM) is to enhance patient quality of life through relief of limiting heart failure symptoms.^1–9^ Historically, in patients who fail to respond to conventional drug therapy with beta-blockers or calcium channel blockers, second-line therapy with disopyramide and/or septal reduction therapies has been recommended, with the latter reserved largely for patients with more-severe symptoms.^10^ However, an enormous diversity in the severity of functional limitation is seen in HCM, underscored by an expanded New York Heart Association (NYHA) functional class II designation that comprises the majority of symptomatic patients with this disease.^11–14^ Indeed, mild symptoms may persist with first-line drug therapy due to suboptimal gradient reduction that may fail to meet a patient’s expectations for additional clinical improvement.^15–17^ Consequently, these individuals—often young and active—must bear a considerable impairment in their daily activities and quality of life that is not adequetly captured by NYHA classification.^8,9,14^ In addition, oHCM patients with class II symptoms remain at risk for development of progressive symptoms and HCM related adverse events.^2,12,13^ Therefore, the opportunity to benefit this specific subgroup of patients with effective therapeutic options remains an important unmet clinical treatment need in oHCM.

Aficamten is a novel cardiac myosin inhibitor (CMI) that reduces or resolves left ventricular outflow tract gradients (LVOT-G) by decreasing the number of actin-myosin cross-bridges and mitigating left ventricular hypercontractility.^16,18^ SEQUOIA-HCM (NCT05186818) was a phase 3 pivotal clinical trial that demonstrated treatment with aficamten is well tolerated and results in improvements across a number of clinically relevant outcomes in patients with oHCM.^16^ However, whether patients with oHCM and mild symptom burden at baseline experience similar benefits with aficamten as those with more severe symptom burden has not been described. Therefore, we sought to explore the efficacy and safety of aficamten in patients in SEQUOIA-HCM according to symptom severity.

## Methods

### Study design

SEQUOIA-HCM was a pivotal phase 3, international, double-blind, randomized, placebo-controlled trial, the design of which has been previously published.^16^ Briefly, 282 adults (18 to 85 years old) with symptomatic oHCM (NYHA functional class II or III; LVOT-G ≥ 30 mmHg at rest and ≥50 mmHg following Valsalva manoeuvre), and reduced exercise capacity (peak oxygen uptake [pVO_2_] ≤ 90% predicted) were randomized 1:1 to either aficamten or placebo for 24 weeks. Patients were anticipated to continue stable doses of their individually optimized background therapy (beta-blockers, non-dihydropyridine calcium channel blockers, and/or disopyramide) for the duration of the study. At 2-week intervals during the first 8 weeks, patients underwent masked echocardiographic-guided dose selection based on site-read left ventricular ejection fraction (LVEF) and Valsalva LVOT-G in order to achieve optimal haemodynamic response (post-Valsalva LVOT-G < 30 and LVEF ≥55%). Full details of the study design and protocol have been described previously.^16^

The trial was approved by the regulatory agencies in the participating countries and by the institutional review board or independent ethics committee at each trial centre. Representatives of Cytokinetics and the steering committee conducted interval reviews of the blinded safety and selected efficacy data. Only an independent Data Monitoring Committee had access to and periodically reviewed unblinded safety data to assess risk to patients during the conduct of the trial. The investigators worked with the sponsor, who performed all statistical analysis, which was also independently confirmed by the Brigham and Women’s Hospital Clinical Trials Outcomes Centre. All patients provided informed consent, and the study was carried out in accordance with the provisions of the Declaration of Helsinki and the International Conference on Harmonization Good Clinical Practice guidelines. The first author (M.S.M.) wrote the paper while all the authors participated in interpretation of the data, critical review of the manuscript, and the decision to submit the manuscript for publication.

### Definition of symptom groups

The primary objective of this study was to characterize the impact of aficamten on clinically relevant outcome measures in patients with relatively mild vs. more-severe disease symptoms, as defined by symptom burden at baseline. The long-standing metric for assessing symptom burden in HCM has been the physician-assigned NYHA functional class. More recently, patient-reported symptom burden using tools such as the Kansas City Cardiomyopathy Questionnaire (KCCQ) has been shown to provide a detailed assessment from the patient’s perspective of the impact of heart failure symptoms on a variety of health status domains (ie, symptom burden, physical limitation, social limitation, and quality of life).^17^ In addition, the KCCQ score has been shown to have greater sensitivity for quantifying symptom burden in HCM, permitting the opportunity to more precisely define patients who are otherwise designated within the broad NYHA classes.^14^ For this reason, we elected to combine both NYHA and KCCQ to define study groups according to both the physicians’ and patients’ perspective of symptom severity at baseline. Mild symptoms were defined as NYHA class II and KCCQ Clinical Summary Score (KCCQ-CSS) ≥ 80 (*n* = 62), and moderate to severe symptoms were defined as NYHA class II/III/IV and KCCQ-CSS <80 (*n* = 71). Based on data from prior large heart failure registries, we applied a KCCQ-CSS cut-off of ≥80, which has been demonstrated to be associated with mild symptoms in patients with heart failure.^19^ The remaining patients (*n* = 14; 9 assigned to aficamten and 5 to placebo) who were assigned to NYHA class III but reported low symptom burden (KCCQ-CSS 80) were excluded from the analysis as they could not confidently be assigned to any group. To assess the impact of excluding these patients, a sensitivity analysis was performed by including them in both symptom groups, and no differences in outcomes were observed (see Supplementary data online, *Tables S1* and *S2*).

### Randomisation

Eligible patients were randomly assigned in a 1:1 ratio to receive aficamten or placebo by an Interactive Web Response System (IWRS). Randomisation was stratified by use of beta-blockers (yes or no) and Cardiopulmonary Exercise Test (CPET) exercise modality (treadmill or bicycle) and implemented in the IWRS. The proportion of patients taking beta-blockers and disopyramide was capped at ∼70% and 10% of the total enrolment, respectively, whereas patients with persistent atrial fibrillation at screening and the number of patients using the bicycle CPET exercise modality were capped at ∼15% and 50%, respectively.

Aficamten was administered orally at 5 mg once daily with three subsequent opportunities to undergo dose increase in 5-mg increments (at Weeks 2, 4, and 6), to a maximum dose of 20 mg, and according to site-read echocardiography measurements. As an additional measure to reduce potential bias, the site investigator and study team were masked to the echocardiogram images and results. Analyses of echocardiographic data were based on values from a central core laboratory blinded to treatment assignment.

Patients were evaluated every 2 or 4 weeks during the 24-week treatment period. CPET assessments were done at screening and Week 24 (end of treatment). Resting transthoracic echocardiography, electrocardiograms, safety laboratory testing, and clinical assessment were collected at each study visit.

### Outcomes

The primary endpoint was change in pVO_2_ from baseline to Week 24. Pre-specified secondary endpoints included changes from baseline to Week 24 in NYHA class, KCCQ-CSS, resting and provocable LVOT-G as well as the cardiac biomarker N-terminal pro–B-type natriuretic peptide (NT-proBNP) and cardiac morphology/function. The two symptom groups randomized to receive aficamten were compared with their respective placebo groups.

NYHA functional class was assigned by the study physician according to the standard NYHA class designation, with an improvement of at least one functional class considered a clinically meaningful change. Patient-reported health status was measured using the 23-item patient-reported outcome measure of KCCQ. The KCCQ-CSS, which is a composite of the total symptom and physical limitation summary scores, was a pre-specified secondary outcome of SEQUOIA-HCM and uses a 23-item score that categorizes symptoms, function and quality of life. Changes in the KCCQ-CSS score of 5, 10, and 20 points, respectively, are associated with clinically important small, moderate to large, and large to very large within-patient changes. Per protocol, NYHA functional class and KCCQ were assessed before other study procedures at each visit, including the end-of-study visit 4 weeks after study medication washout.

### Statistical analysis

Baseline characteristics were summarized using means and standard deviation for normally distributed continuous variables, medians and interquartile range for right-skewed variables, and counts with percentages for categorical variables. Differences in baseline characteristics between the patients with oHCM and mild symptoms and those with moderate to severe symptoms were compared using *t*-tests. Wilcoxon rank-sum tests, and Pearson’s χ² test, as appropriate. Within-group changes from baseline to 24 were assessed using paired *t*-tests. Treatment effect estimates were analysed using linear regression, adjusted for baseline values of continuous outcomes, randomized treatment, and stratification variables (beta-blocker use and CPET modality). NTproBNP values were log-transformed prior to analyses and are reported as proportional changes. Differences in treatment effect by symptom severity group were tested with interaction terms. *P*-values <.05 were considered statistically significant. Owing to the *post hoc* nature of these analyses, no adjustment was made for multiple testing. Given the potential for false positive findings in the presence of multiple testing, all findings should be considered hypothesis-generating. Statistical analyses were conducted using STATA version 18.5 (StataCorp, College Station, TX).

## Results

### Baseline characteristics

A total of 118 patients had mild symptoms at baseline (of whom 62 were randomized to aficamten), and 150 patients had moderate to severe symptoms at baseline (of whom 71 were randomized to aficamten). At baseline, the proportion of females, patient’s race, geographic region of recruiting centre, and body mass index were different between the two symptom groups, whereas all other baseline characteristics, including clinical and imaging variables, were similar (*Table 1*, Supplementary data online, *Tables S3* and *S4*).

**Table 1:** Demographic and clinical characteristics of patients with obstructive HCM and mild vs. moderate to severe symptoms

	Mild symptoms^a^ *n* = 118	Moderate to severe symptoms^b^ *n* = 150	*P-*value
Randomized to active treatment	62 (53)	71 (47)	0.40
Age, y	58 ± 13	60 ± 12	0.29
Female sex	36 (31)	72 (48)	0.004
Race			<0.001
Asian	37 (31)	15 (10)	
Black or African American	2 (2)	1 (1)	
Other	2 (2)	0 (0)	
White	77 (65)	134 (89)	
Geographic region			<0.001
China	31 (26)	13 (9)	
North America	28 (24)	58 (39)	
Rest of world	59 (50)	79 (53)	
Medical history			
Hypertension	57 (48)	83 (55)	0.25
Known HCM-causing gene mutation	15 (13)	30 (20)	0.11
Positive family history of HCM	28 (24)	44 (29)	0.30
Paroxysmal atrial fibrillation	14 (12)	25 (17)	0.27
Coronary artery disease	15 (13)	16 (11)	0.60
Diabetes	12 (10)	11 (7)	0.41
Permanent atrial fibrillation	2 (2)	1 (1)	0.43
Vital signs			
Systolic blood pressure, mmHg	125 ± 14	125 ± 17	0.77
Diastolic blood pressure, mmHg	75 ± 10	74 ± 11	0.51
Resting heart rate, bpm	68 ± 12	71 ± 13	0.08
BMI, kg/m^2^	27 ± 4	29 ± 4	0.003
Background HCM therapy			
Beta-blocker	67 (57)	94 (63)	0.33
Calcium channel blocker	41 (35)	55 (37)	0.74
Selective calcium channel blockers with direct cardiac effects	32 (27)	48 (32)	0.39
Selective calcium channel blockers with mainly vascular effects	10 (9)	7 (5)	0.20
Disopyramide	13 (11)	21 (14)	0.47
ICD	15 (13)	21 (14)	0.76
KCCQ-CSS	90 ± 6	61 ± 14	<0.001
NYHA functional class			<0.001
II	118 (100)	96 (64)	
III	0 (0)	53 (35)	
IV	0 (0)	1 (1)	
NT-proBNP, pg/mL	806 [317–1613]	676 [344–1737]	0.97
hsTroponin I type 3, ng/L	16 [8–38]	10 [7–22]	0.011
CPET			
CPET modality: Bicycle	58 (49)	62 (41)	0.20
CPET modality: Treadmill	60 (51)	88 (59)	0.20
Total workload CPET, watts	127 ± 39	119 ± 41	0.10
pVO_2_ CPET, mL/kg/min	19 ± 4	18 ± 4	0.011
% Predicted oxygen uptake	58 ± 12	56 ± 11	0.14
Peak respiratory exchange ratio	1 ± 0	1 ± 0	0.49
Echocardiographic parameters			
Valsalva LVOT-G, mmHg	81.4 ± 33.5	84.3 ± 31.4	0.46
LVEF, %	75.0 ± 6.0	74.7 ± 5.9	0.68
Resting LVOT-G, mmHg	54.0 ± 29	55.9 ± 30.6	0.61
LV maximal wall thickness, cm	2.1 ± 0.3	2.1 ± 0.3	0.12
BSA indexed LAV, mL/m^2^	39.7 ± 12	40.7 ± 15.3	0.58
Septal E/e′	19.5 ± 9.1	20.1 ± 8.1	0.61
Lateral E/e′	15.5 ± 7.9	15.6 ± 7.2	0.86

^a^NYHA class II and KCCQ-CSS ≥80.

^b^NYHA class II/III/IV and KCCQ-CSS <80.

Data are *n* (%), mean ± SD, or median [range].

BMI, body mass index; BSA, body surface area; CPET, cardiopulmonary exercise test; E/e′, peak E wave to annular early diastolic velocity ratio; HS, high sensitivity; ICD, Implantable cardioverter defibrillator; KCCQ-CSS, Kansas City Cardiomyopathy Questionnaire-Clinical Summary Score; LAV, left atrial volume; LV, left ventricular; LVEF, left ventricular ejection fraction; LVOT-G, left ventricular outflow tract gradient; NT-proBNP, N-terminal pro-B-type natriuretic peptide; NYHA, New York Heart Association; pVO_2_, peak oxygen uptake.

### Functional improvement

**Figure 1:**
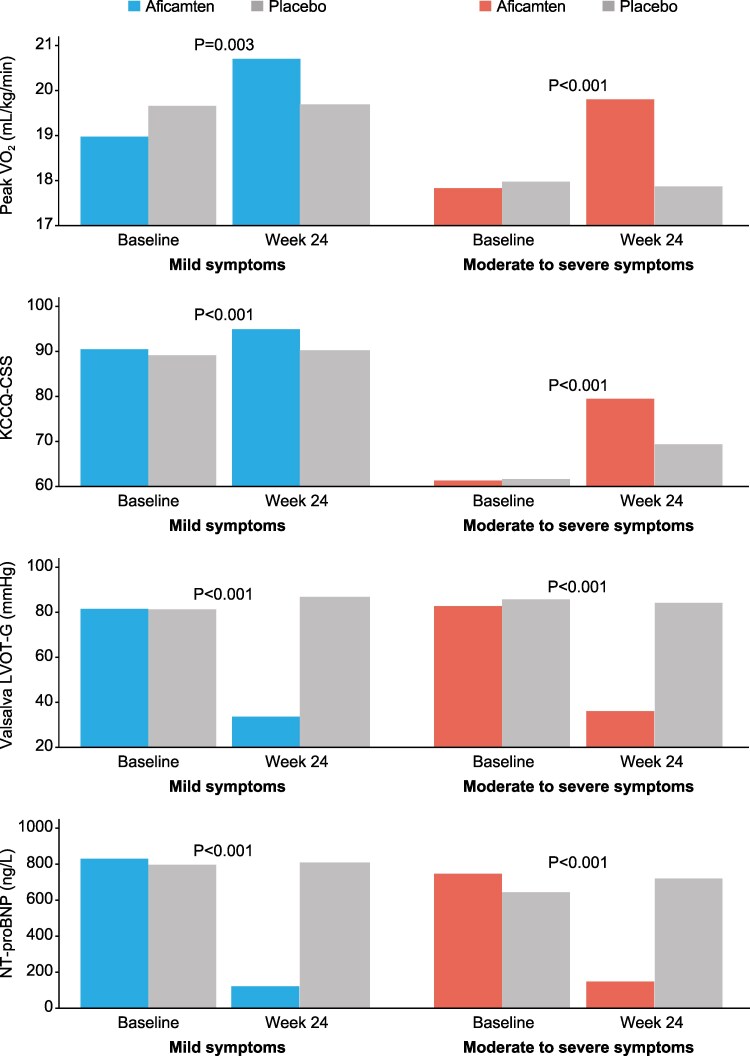
Key Primary and secondary outcomes in patients with symptomatic oHCM treated with aficamten. Shown are mean changes from BL to Week 24 in (*A*) pVO_2_, (*B*) KCCQ-CSS, (*C*) Valsalva LVOT-G, and (*D*) NT-proBNP in patients with oHCM and mild vs. moderate to severe symptoms treated with aficamten vs. placebo. *P* values refer to the change in outcome from baseline to Week 24 in patients with oHCM and mild or moderate to severe symptoms treated with aficamten as compared with placebo. BL, baseline; KCCQ-CSS, Kansas City Cardiomyopathy Questionnaire-Clinical Summary Score; LVOT-G, left ventricular outflow tract gradient; NT-proBNP, N-terminal pro–B-type natriuretic peptide; oHCM, obstructive hypertrophic cardiomyopathy; pVO_2_, peak oxygen uptake

From baseline to week 24, pVO_2_ increased from 19.0 mL/kg/min to 20.7 mL/kg/min [95% confidence interval (CI): 0.8 to 2.6] in aficamten-treated patients with mild symptoms (*P* = .003 vs. placebo) and from 17.8 mL/kg/min to 19.8 mL/kg/min (95% CI: 1.2 to 2.6) in aficamten-treated patients with moderate to severe symptoms (*P* < .001 vs. placebo) (*Figure 1*). The treatment effect of aficamten on pVO_2_ was similar between the two symptom groups [change (Δ) of 1.6 mL/kg/min (95% CI: 0.5 to 2.7) in mild symptoms group vs. Δ1.8 mL/kg/min (95% CI: 0.8 to 2.8) in moderate to severe symptoms group; *P* = .83 for interaction] (*Table 2*, *Structured Graphical Abstract*).

**Table 2:** Comparisons of aficamten treatment effect and safety outcomes in oHCM patients with mild vs. moderate to severe symptoms

	Symptom groups		
	Mild symptoms^a^ *n* = 118	Moderate to severe symptoms^b^ *n* = 150	
Efficacy outcomes	Treatment effect mean (95% CI)	Treatment effect mean (95% CI)	Interaction *P*-value
pVO_2_ change at Week 24, mL/kg/min	1.6 (0.5, 2.7)	1.8 (0.8, 2.8)	0.83
KCCQ-CSS change at Week 24	4 (1, 6)	10 (6, 15)	0.02
≥1 NYHA functional class improvement at Week 24, %	34 (16, 49)	57 (19, 49)	0.92
Valsalva LVOT-G change at Week 24, mmHg	−53 (−62, −44)	−47 (−57, −37)	0.43
Resting LVOT-G change at Week 24, mmHg	−41 (−49, −33)	−38 (−47, −29)	0.64
Valsalva LVOT-G < 30 mmHg at Week 24, %	54 (40, 68)	37 (25, 50)	0.07
NT-proBNP change at Week 24, %	−79 (−83, −73)	−81 (−85, −76)	0.56
LA volume index change at Week 24, mL/m^2^	−4.6 (−7.3, −1.9)	−3.3 (−5.5, −1.2)	0.45
Septal E/e′ change at Week 24	−4.3 (−6.4, −2.3)	−3.1 (−4.2, −1.9)	0.20
Lateral E/e′ change at Week 24	−3.4 (−5.1, −1.8)	−3.4 (−5.1, −1.7)	0.97
Maximum LV wall thickness change at Week 24, cm	−0.16 (−0.25, −0.07)	−0.11 (−0.18, −0.03)	0.38
Safety outcomes	**Aficamten vs. placebo, *n* (%)**		
Any adverse event (TE)	49 (79) vs. 35 (63)	51 (72) vs. 61 (77)	–
Any serious adverse event (TE)	0 (0) vs. 5 (9)	8 (11) vs. 8 (10)	–
LVEF <50%	3 (5) vs. 0 (0)	2 (3) vs. 1 (1)	–

^a^NYHA class II and KCCQ-CSS ≥80.

^b^NYHA class II/III/IV and KCCQ-CSS <80.

CI, confidence interval; E/e′, peak E wave to annual diastolic velocity ratio; KCCQ-CSS, Kansas City Cardiomyopathy Questionnaire-Clinical Summary Score; LA, left atrial; LV, left ventricular; LVEF, left ventricular ejection fraction; LVOT-G, left ventricular outflow tract gradient; NT-proBNP, N-terminal pro–B-type natriuretic peptide; NYHA, New York Heart Association; oHCM, obstructive hypertrophic cardiomyopathy; pVO_2_, peak oxygen uptake; TE, treatment-emergent.

Among the patients with oHCM and mild symptoms treated with aficamten, a small improvement in pVO_2_ from 0 to 1.5 mL/kg/min was observed in 13 (22%) patients (44% with <1.0 mL/kg/min), a moderate improvement ≥1.5 to 3 mL/kg/min in 11 (19%) patients, a large improvement ≥3 mL/kg/min in 15 (25%) patients, and no improvement or worsening in 20 (34%) patients. For each pVO_2_ category, the proportion of patients with improvement was similar to that observed for aficamten-treated patients with moderate to severe symptoms (22% small vs. 23% moderate vs. 32% large vs. 23% no improvement; *P* = .6 for interaction). In addition, the proportion of aficamten-treated patients who had a ≥ 1.5 mL/kg/min improvement in pVO_2_ and an improvement of at least one NYHA functional class at Week 24 was similar for those with mild vs. moderate to severe symptoms [*n* = 16 (26%) vs. *n* = 24 (34%); *P* = .3 for interaction]. Likewise, the proportion of aficamten-treated patients (mild vs. moderate to severe symptoms) who had a ≥ 3.0 mL/kg/min improvement in pVO_2_ and no worsening in NYHA functional class [*n* = 15 (24%) vs. *n* = 21 (30%); *P* = .5], or both a ≥ 3.0 mL/kg/min improvement in pVO_2_ and an improvement of at least one NYHA functional class [*n* = 7 (11%) vs. *n* = 13 (18%); *P* = .3 for interaction] was similar between symptom groups (*Table 3*).

**Table 3:** Comparison of changes in symptoms and functional capacity in patients with oHCM and mild vs. moderate to severe symptoms treated with aficamten

	Mild symptoms^a^ *n* = 62	Moderate to severe symptoms^b^ *n* = 71	Interaction *P*-value
≥1.5 mL/kg/min increase in pVO_2_ and ≥1 NYHA functional class improvement	16 (26)	24 (34)	0.3
≥3.0 mL/kg/min increase in pVO_2_ and no worsening of NYHA functional class	15 (24)	21 (30)	0.5
≥3.0 mL/kg/min increase in pVO_2_ and ≥1 NYHA functional class improvement	7 (11)	13 (18)	0.3

^a^NYHA class II and KCCQ-CSS ≥80.

^b^NYHA class II/III/IV and KCCQ-CSS <80.

Data are *n* (%).

KCCQ-CSS, Kansas City Cardiomyopathy Questionnaire-Clinical Summary Score; NYHA, New York Heart Association; pVO_2_, peak oxygen uptake.

### Impact of aficamten on secondary end points

#### Health status by KCCQ-CSS

Over the treatment period, KCCQ-CSS improved with aficamten treatment in both symptom groups, from 90 ± 6 to 95 ± 6 in the mild symptoms group (*P* = .004 vs. placebo) and from 61 ± 15 to 80 ± 18 in the moderate to severe symptoms group (*P* < .001 vs. placebo) (*Figure 1*). The treatment effect was greater in patients with moderate to severe symptoms at baseline [Δ + 10 (95% CI: 6 to 15) vs. Δ + 4 (95% CI: 1 to 6); interaction *P* = .02] (*Table 2*, *Structured Graphical Abstract*).

In the mild symptom group treated with aficamten, 12 (20%) patients had a small but clinically important improvement from 5 to 9 points in KCCQ-CSS and 14 (23%) had a moderate to large improvement from 10 to 19 points. In addition, the remaining 34 (57%) patients had a < 5-point change (baseline KCCQ-CSS of 93). Among patients treated with aficamten in the moderate to severe symptoms group, 6 (9%) had a small improvement in KCCQ-CSS, 18 (26%) a moderate to large change, 31 (44%) had a very large improvement, and 14 (20%) had a < 5 point change (*P* < .001).

#### NYHA functional class

At Week 24, 33 (53%) of 62 patients with oHCM and mild symptoms treated with aficamten had an improvement of one NYHA functional class (i.e., transitioned to class I; *P* < .001 vs. placebo), which was a similar magnitude of improvement compared with patients with moderate to severe symptoms treated with aficamten [*n* = 41 (57%) of 71; *P* = .92 for interaction] (*Table 2*).

**Figure 2:**
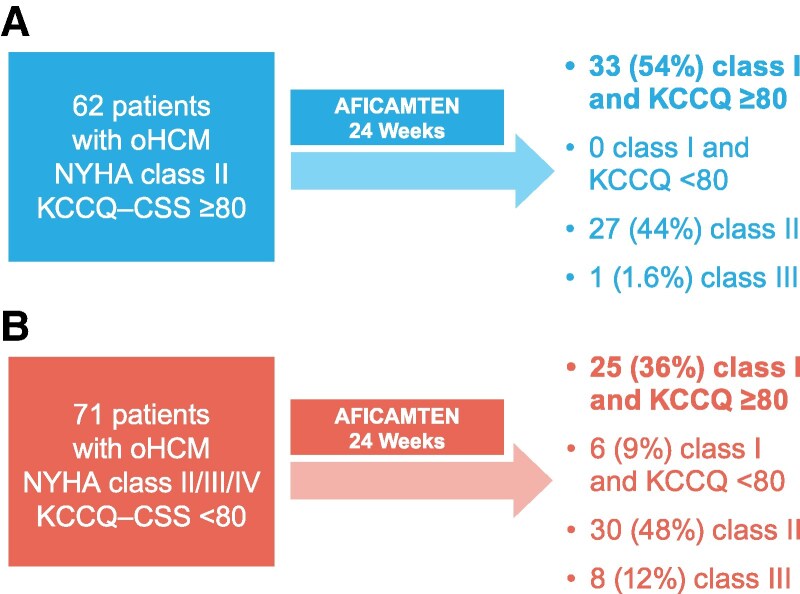
Changes in symptom burden and health status from baseline to Week 24. Transition in NYHA functional class and KCCQ-CSS from BL to completion of the 24-week treatment period in aficamten-treated patients with (*A*) mild symptoms and (*B*) moderate to severe symptoms. BL, baseline; KCCQ-CSS, Kansas City Cardiomyopathy Questionnaire-Clinical Summary Score; NYHA, New York Heart Association; oHCM, obstructive hypertrophic cardiomyopathy

At the end of treatment, 33 (54%) aficamten-treated patients in the mild symptoms group were NYHA class I and KCCQ-CSS ≥80, none were class I and KCCQ-CSS <80, 27 (44%) patients remained class II, and 1 (1.6%) had worsening to class III. By contrast, 25 (36%) aficamten-treated patients in the moderate to severe symptoms group were NYHA class I and KCCQ-CSS ≥80, 6 (9%) were class I and KCCQ-CSS <80, 30 (48%) class II, and 8 (12%) class III (*P* = .7) (*Figure 2*).

### Relationship between LVOT-G, symptoms, and cardiac structure

Compared with baseline, resting and Valsalva LVOT-G, respectively, in aficamten-treated patients decreased at 24 weeks from 54 ± 30 mmHg to 19 ± 16 mmHg and 82 ± 37 mmHg to 34 ± 23 mmHg in the mild symptoms group (both *P* < .001 vs. placebo) and from 55 ± 24 mmHg to 20 ± 17 mmHg and 83 ± 27 mmHg to 36 ± 25 mmHg in the moderate to severe symptoms group (both *P* < .001 vs. placebo) (*Figure 1*).

The treatment effect of aficamten on resting and provocable outflow gradients was similar in patients with mild vs. moderate to severe symptoms [resting LVOT-G: Δ −41 mmHg (95% CI: −49 to −33) vs. Δ −38 mmHg (95% CI: −47 to −29), *P*-interaction = .64; Valsalva LVOT-G: Δ −53 mmHg (95% CI: −62 to −44) vs. Δ −47 mmHg (95% CI: −57 to −37), *P* = .43] (*Table 2*, Graphical Abstract). A complete haemodynamic response (resting <30 mmHg and Valsalva <50 mmHg) was achieved in 46 (74%) aficamten-treated patients with mild symptoms vs. 44 (62%) with moderate to severe symptoms (*P* = .13). In addition, measures of cardiac remodelling, including a decrease in left atrial volume index and maximal left ventricular wall thickness from baseline to Week 24 were similar between the two aficamten-treated symptom groups (*Table 2*).

### Biomarkers

The treatment effect of aficamten on magnitude of change in serum concentration of NT-proBNP from baseline to Week 24 was similar in patients with oHCM and mild symptoms limitation compared with patients with moderate to severe symptoms limitation [Δ −79% (95% CI: −83 to −73) vs. Δ −81% (95% CI: −85 to −76); *P* = .56 for interaction] (*Table 2*). Among patients with mild symptoms randomized to aficamten, 52 (84%) of 62 had a > 50% relative reduction in NT-proBNP concentration at the end of treatment, which was similar to patients with moderate to severe symptoms randomized to aficamten (56 79% of 71; *P* = .5 for interaction).

### Safety

At Week 24 in aficamten-treated patients, the LVEF in the mild symptoms group was not significantly different than the moderate to severe symptoms group (least squares mean difference, +0.7% points; 95% CI: −1.8 to +3.1). Among aficamten-treated patients, a transient reduction to LVEF <50% occurred in 3 (5%) patients with mild symptoms and in 2 (3%) with moderate to severe symptoms (*Table 2*), none of whom underwent a treatment interruption or developed clinical heart failure. In the mild symptoms group, none of the patients treated with aficamten and 5 (9%) treated with placebo had serious adverse events. In the moderate to severe symptoms group, 8 (11%) patients treated with aficamten and 8 (10%) treated with placebo had serious adverse events.

## Discussion

The majority of patients with HCM have the propensity to develop LVOT obstruction,^20^ which represents the most powerful determinant of limiting symptoms in this disease.^21^ However, an enormous diversity in the severity of limiting symptoms is seen in HCM, not adequately captured by the NYHA classification underscored by an expanded functional class II designation that comprises most patients who are symptomatic.^11–14,22,23^ Within the broad NYHA II symptom class are patients with oHCM who experience mild symptoms despite conventional drug therapy with beta-blockers or calcium channel blockers,^2–4^ a consequence of suboptimal LVOT-G lowering and increased risk for future progressive heart failure symptoms.^2–4,15,16^

The present observations from SEQUOIA-HCM support a potential clinical benefit of CMI therapy in patients with oHCM who continue to experience mild symptoms burden with impaired quality of life, despite standard background therapy. Indeed, in this subgroup of patients with oHCM, representing over 40% of the trial population, treatment with aficamten achieved substantial improvement across several clinically meaningful outcome measures, including symptom relief, improved exercise capacity, and reduced NT-proBNP concentration. Notably, the magnitude of treatment benefit in patients with mild symptoms was similar to that observed in patients with more advanced symptoms. Furthermore, at the end of a relatively short treatment period of 24 weeks, more than half of the patients with mild symptoms who were treated with aficamten became asymptomatic with restoration to normal quality of life and full engagement in daily activities of life (NYHA class I and KCCQ-CSS ≥80). In addition, a substantial proportion of these patients had a moderate to large enhancement in health status of 10 or more points by the KCCQ, despite demonstrating relatively high baseline KCCQ scores.

These health status benefits observed with aficamten were supported by measures of function, including improvements in exercise capacity with pVO_2._ Notably, over 24 weeks of aficamten treatment, mildly symptomatic patients with oHCM experienced an average increase in pVO_2_ of 1.6 mL/kg/min. Whether this degree of improvement will be associated with improved outcome over an extended follow-up period is unknown and will require further studies to clarify. However, it is notable that this increase in exercise capacity with aficamten exceeded what is considered to represent a clinically meaningful change in pVO_2_ of 1.0 mL/kg/min, a cut-off independently associated with reduced risk of heart failure death and transplant in HCM.^24^ Furthermore, improvements in exercise capacity smaller than those achieved in this substudy of the SEQUOIA-HCM trial have been associated with enhanced survival in non-HCM heart failure populations.^25^ The magnitude of improvement, across different pVO_2_ values observed in our patients, was also similar between symptom groups.

These observations support the emerging principle that the clinical benefit of aficamten is independent of symptom burden, reflecting the marked haemodynamic effects observed across the whole spectrum of obstructive disease. Indeed, three-quarters of the patients with oHCM and mild symptoms experienced a near complete relief of outflow tract obstruction (resting LVOT-G < 30 mmHg and Valsalva LVOT-G < 50 mmHg) with aficamten, resulting in normalisation (or near normalisation) of left ventricular cavity pressures. In addition, patients in the mild symptoms group demonstrated other favourable effects of aficamten, including a 50% reduction in serum NT-proBNP concentration and potentially favourable cardiac remodelling with a decrease in left atrial size and left ventricular wall thickness and an improvement in measures of diastolic function. Whether these changes in cardiac biomarker and morphology are independently associated with favourable disease-related outcomes, beyond improvements in measures of feel and function, will require longer follow-up to resolve. Nevertheless, persuasive observational evidence from the long-standing surgical experience in HCM with myectomy supports the principle that by abolishing subaortic obstruction, natural history of this disease is favourably improved.^5,6,26^

The clinical benefit demonstrated by CMI drug therapy in patients with oHCM and a level of symptom limitation less marked than what has been traditionally considered for more potent drug therapy has raised the prospect for a revised treatment narrative. Indeed, the option of earlier medical therapy with CMI offers a potential opportunity for patients to experience extended periods without significant impairment in quality of life. Conversely, continuing to delay such therapeutic intervention may possibly subject patients to potentially diminishing functional capacity progressing to more advanced symptom limitation despite first-line drug therapy with atrial ventricular nodal blocking agents or disopyramide.^2–4,21,27^ As an alternative, given the proven efficacy and low risk of septal myectomy,^5,6^ recent HCM guidelines have recommended surgery in patients with less symptom burden than traditionally considered, particularly in patients with specific high risk features (e.g. severe pulmonary hypertension, young patient with high rest gradients, and left atrial enlargement with paroxysmal atrial fibrillation).^8^ However, whether myectomy (or, alternatively, alcohol septal ablation) will have a comparable clinical benefit if performed in patients with oHCM and a similar mild symptom burden as demonstrated in this study is uncertain.

It is notable that the robust gradient reduction and resultant clinical benefit in the mild symptoms group treated with aficamten was achieved with only modest change in systolic function and no interruptions in treatment for the small number of patients who had transient LVEF <50% (3%), none of whom had clinical heart failure, underscoring a favourable benefit-to-risk ratio achieved by reversing limiting symptoms and improving health status beyond that achieved with first-line drug therapy.

There were several limitations in this study. First, the relatively short study treatment period limits the opportunity to determine if patients may derive a greater level of clinical benefit from longer duration of exposure to aficamten therapy, including the 44% of patients with mild symptoms who did not achieve a clinically relevant improvement of ≥1 mL/kg/min in pVO_2_. We anticipate having additional insights regarding this issue with the FOREST-HCM study, which is evaluating aficamten in patients with HCM over an extended follow-up period. Second, in order to distinguish the two study groups with different symptom burden, we chose a cut-off of ≥80 points for KCCQ to further classify patients as having mild symptoms. However, we recognize that even patients with KCCQ scores below this threshold may have symptoms considered, by the patient, to be relatively mild.^28,29^ In this regard, we may have underestimated the magnitude of potential treatment benefit of aficamten in this subgroup defined as mildly symptomatic. Finally, we observed an important placebo effect in this study, similar to that seen in other HCM drug therapy studies.^15^ The mechanisms responsible for changes associated with placebo treatment are likely complex but include differences in how patients with HCM perceive impact of their chronic and often progressive disease on daily symptoms as well as the unique pathophysiology of HCM in which outflow obstruction can be substantially influenced by alterations in loading conditions and contractility and therefore impacted by changes in patients lifestyle or behaviours.

In conclusion, patients with oHCM and mild symptoms have historically represented an unmet treatment need within the diverse and heterogenous HCM disease spectrum, particularly given the generally low efficacy of and potential for side effects related to beta-blockers and calcium channel blockers. In this study, the addition of aficamten to standard of care drug therapy in this subgroup of patients with oHCM appeared safe and efficacious with a significant treatment benefit across a range of clinically relevant outcomes and generally comparable to that observed in more symptomatic patients. Therefore, it may be reasonable to consider earlier treatment with aficamten in patients with oHCM, without waiting for the development of marked disability. In addition, these data support the need for further investigations characterizing the impact of even earlier treatment intervention with aficamten on natural history of disease progression in patients with oHCM.

## Supplementary Material

ehaf364_Supplementary_Data

## Declarations

### Data Availability

Qualified researchers may submit a request containing the research objectives, endpoints/outcomes of interest, statistical analysis plan, data requirements, publication plan, and qualifications of the researcher(s). Requests are reviewed by a committee of internal and external advisers. If approved, data necessary to address the research question will be provided under the terms of the data sharing agreement. Requests will be considered after applications for marketing authorisation in the US and Europe have been reviewed and final decisions rendered. Requests may be submitted to medicalaffairs@cytokinetics.com.

### Pre-registered Clinical Trial Number

SEQUOIA-HCM (NCT05186818).

## References

[1] Maron BJ . Clinical course and management of hypertrophic cardiomyopathy. N Engl J Med 2018;379:655–68. 10.1056/NEJMra1710575 30110588

[2] Sherrid MV, Shetty A, Winson G, Kim B, Musat D, Alviar CL, et al Treatment of obstructive hypertrophic cardiomyopathy symptoms and gradient resistant to first-line therapy with beta-blockade or verapamil. Circ Heart Fail 2013;6:694–702. 10.1161/CIRCHEARTFAILURE.112.000122 23704138

[3] Dybro AM, Rasmussen TB, Nielsen RR, Andersen MJ, Jensen MK, Poulsen SH. Randomized trial of metoprolol in patients with obstructive hypertrophic cardiomyopathy. J Am Coll Cardiol 2021;78:2505–17. 10.1016/j.jacc.2021.07.065 34915981

[4] Adler A, Fourey D, Weissler-Snir A, Hindieh W, Chan RH, Gollob MH, et al Safety of outpatient initiation of disopyramide for obstructive hypertrophic cardiomyopathy patients. J Am Heart Assoc 2017;6. 10.1161/JAHA.116.005152 PMC5669159 28550094

[5] Kotkar KD, Said SM, Dearani JA, Schaff HV. Hypertrophic obstructive cardiomyopathy: the mayo clinic experience. Ann Cardiothorac Surg 2017;6:329–36. 10.21037/acs.2017.07.03 28944173 PMC5602208

[6] Ommen SR, Maron BJ, Olivotto I, Maron MS, Cecchi F, Betocchi S, et al Long-term effects of surgical septal myectomy on survival in patients with obstructive hypertrophic cardiomyopathy. J Am Coll Cardiol 2005;46:470–6. 10.1016/j.jacc.2005.02.090 16053960

[7] Veselka J, Jensen MK, Liebregts M, Januska J, Krejci J, Bartel T, et al Long-term clinical outcome after alcohol septal ablation for obstructive hypertrophic cardiomyopathy: results from the euro-ASA registry. Eur Heart J 2016;37:1517–23. 10.1093/eurheartj/ehv693 26746632

[8] Writing Committee M, Ommen SR, Ho CY, Asif IM, Balaji S, Burke MA, et al 2024 AHA/ACC/AMSSM/HRS/PACES/SCMR guideline for the management of hypertrophic cardiomyopathy: a report of the American Heart Association/American College of Cardiology joint committee on clinical practice guidelines. J Am Coll Cardiol 2024;83:2324–405. 10.1016/j.jacc.2024.02.014 38727647

[9] Arbelo E, Protonotarios A, Gimeno JR, Arbustini E, Barriales-Villa R, Basso C, et al 2023 ESC guidelines for the management of cardiomyopathies. Eur Heart J 2023;44:3503–626. 10.1093/eurheartj/ehad194 37622657

[10] Gersh BJ, Maron BJ, Bonow RO, Dearani JA, Fifer MA, Link MS, et al 2011 ACCF/AHA guideline for the diagnosis and treatment of hypertrophic cardiomyopathy: a report of the American College of Cardiology Foundation/American Heart Association Task Force on Practice Guidelines. Circulation 2011;124:e783–831. 10.1161/CIR.0b013e318223e2bd 22068434

[11] Maron BJ, Rowin EJ, Maron MS. Global burden of hypertrophic cardiomyopathy. JACC Heart Fail 2018;6:376–8. 10.1016/j.jchf.2018.03.004 29724362

[12] Ho CY, Day SM, Ashley EA, Michels M, Pereira AC, Jacoby D, et al Genotype and lifetime burden of disease in hypertrophic cardiomyopathy: insights from the sarcomeric human cardiomyopathy registry (SHaRe). Circulation 2018;138:1387–98. 10.1161/CIRCULATIONAHA.117.033200 30297972 PMC6170149

[13] Maurizi N, Olivotto I, Maron MS, Bonacchi G, Antiochos P, Tomberli B, et al Lifetime clinical course of hypertrophic cardiomyopathy: outcome of the historical Florence cohort over 5 decades. JACC Adv 2023;2:100337. 10.1016/j.jacadv.2023.100337 38938243 PMC11198069

[14] Sherrod C, Spertus JA, Gosch KL, Wang A, Elliott PM, Lakdawala NK, et al The Kansas city cardiomyopathy questionnaire in relation to New York Heart Association class. J Card Fail 2025;31:481–4. 10.1016/j.cardfail.2024.08.061 39349158 PMC11975531

[15] Olivotto I, Oreziak A, Barriales-Villa R, Abraham TP, Masri A, Garcia-Pavia P, et al Mavacamten for treatment of symptomatic obstructive hypertrophic cardiomyopathy (EXPLORER-HCM): a randomised, double-blind, placebo-controlled, phase 3 trial. Lancet 2020;396:759–69. 10.1016/S0140-6736(20)31792-X 32871100

[16] Maron MS, Masri A, Nassif ME, Barriales-Villa R, Arad M, Cardim N, et al Aficamten for symptomatic obstructive hypertrophic cardiomyopathy. N Engl J Med 2024;390:1849–61. 10.1056/NEJMoa2401424 38739079

[17] Nassif M, Fine JT, Dolan C, Reaney M, Addepalli P, Allen VD, et al Validation of the Kansas city cardiomyopathy questionnaire in symptomatic obstructive hypertrophic cardiomyopathy. JACC Heart Fail 2022;10:531–9. 10.1016/j.jchf.2022.03.002 35902155

[18] Malik FI, Robertson LA, Armas DR, Robbie EP, Osmukhina A, Xu D, et al A phase 1 dose-escalation study of the cardiac myosin inhibitor aficamten in healthy participants. JACC Basic Transl Sci 2022;7:763–75. 10.1016/j.jacbts.2022.04.008 36061336 PMC9436819

[19] Spertus JA, Jones PG, Kim J, Globe D. Validity, reliability, and responsiveness of the Kansas city cardiomyopathy questionnaire in anemic heart failure patients. Qual Life Res 2008;17:291–8. 10.1007/s11136-007-9302-5 18165909 PMC2238779

[20] Maron MS, Olivotto I, Zenovich AG, Link MS, Pandian NG, Kuvin JT, et al Hypertrophic cardiomyopathy is predominantly a disease of left ventricular outflow tract obstruction. Circulation 2006;114:2232–9. 10.1161/CIRCULATIONAHA.106.644682 17088454

[21] Maron MS, Olivotto I, Betocchi S, Casey SA, Lesser JR, Losi MA, et al Effect of left ventricular outflow tract obstruction on clinical outcome in hypertrophic cardiomyopathy. N Engl J Med 2003;348:295–303. 10.1056/NEJMoa021332 12540642

[22] Spertus JA, Fine JT, Elliott P, Ho CY, Olivotto I, Saberi S, et al Mavacamten for treatment of symptomatic obstructive hypertrophic cardiomyopathy (EXPLORER-HCM): health status analysis of a randomised, double-blind, placebo-controlled, phase 3 trial. Lancet 2021;397:2467–75. 10.1016/S0140-6736(21)00763-7 34004177

[23] Sherrod CF, Saberi S, Nassif ME, Claggett BL, Coats CJ, Garcia-Pavia P, et al Effect of aficamten on health status outcomes in obstructive hypertrophic cardiomyopathy: results from SEQUOIA-HCM. J Am Coll Cardiol 2024;84:1773–85. 10.1016/j.jacc.2024.08.014 39217569 PMC11975529

[24] Coats CJ, Rantell K, Bartnik A, Patel A, Mist B, McKenna WJ, et al Cardiopulmonary exercise testing and prognosis in hypertrophic cardiomyopathy. Circ Heart Fail 2015;8:1022–31. 10.1161/CIRCHEARTFAILURE.114.002248 26374874

[25] Swank AM, Horton J, Fleg JL, Fonarow GC, Keteyian S, Goldberg L, et al Modest increase in peak VO2 is related to better clinical outcomes in chronic heart failure patients: results from heart failure and a controlled trial to investigate outcomes of exercise training. Circ Heart Fail 2012;5:579–85. 10.1161/CIRCHEARTFAILURE.111.965186 22773109 PMC3732187

[26] Cui H, Schaff HV, Wang S, Lahr BD, Rowin EJ, Rastegar H, et al Survival following alcohol septal ablation or septal myectomy for patients with obstructive hypertrophic cardiomyopathy. J Am Coll Cardiol 2022;79:1647–55. 10.1016/j.jacc.2022.02.032 35483751

[27] Maron MS, Rowin EJ, Olivotto I, Casey SA, Arretini A, Tomberli B, et al Contemporary natural history and management of nonobstructive hypertrophic cardiomyopathy. J Am Coll Cardiol 2016;67:1399–409. 10.1016/j.jacc.2016.01.023 27012399

[28] Lee MMY, Masri A, Nassif ME, Barriales-Villa R, Abraham TP, Claggett BL, et al Aficamten and cardiopulmonary exercise test performance: a substudy of the SEQUOIA-HCM randomized clinical trial. JAMA Cardiol 2024;9:990–1000. 10.1001/jamacardio.2024.2781 39230885 PMC11375526

[29] Sandhu AT, Zheng J, Kalwani NM, Gupta A, Calma J, Skye M, et al Impact of patient-reported outcome measurement in heart failure clinic on clinician health status assessment and patient experience: a substudy of the PRO-HF trial. Circ Heart Fail 2023;16:e010280. 10.1161/CIRCHEARTFAILURE.122.010280 36334312 PMC10108581

