# Supplementary material for: Efficacy of aficamten in patients with obstructive hypertrophic cardiomyopathy and mild symptoms: results from the SEQUOIA-HCM trial
Source: Eur Heart J. 2025 May 17;46(40):4076–86. doi: 10.1093/eurheartj/ehaf364 (PMC12539928; doi:10.1093/eurheartj/ehaf364)
Supplement: ehaf364_Supplementary_Data [file ehaf364_supplementary_data.pdf]

Supplementary Appendix

Efficacy of Aficamten in Patients With Obstructive Hypertrophic Cardiomyopathy and Mild Symptoms

Martin S. Maron[a], Juan Ramon Gimeno [b], Josef Veselka[c], Roberto Barriales-Villa[d], Brian L. Claggett[e], Caroline J. Coats[f], Sheila M. Hegde[g], James L. Januzzi[h,i], Ian J. Kulac[e], Ahmad Masri[j], Michael E. Nassif[k], John A. Spertus[l], Daniel L. Jacoby[m], Stephen. B. Heitner[m], Stuart Kupfer[m], Fady I Malik[m], Amy Wohltman[m], Iacopo Olivotto[n], on behalf of the SEQUOIA-HCM Investigators

[a]Lahey Hospital and Medical Center, Burlington, MA, USA; [b]Cardiac Department, University Hospital Virgen Arrixaca, CIBERCV, ERN Guard-Heart, Murcia, Spain; [c]Institute of Health Information and Statistics, Prague, Czech Republic; [d]Complexo Hospitalario Universitario A Coruña, INIBIC, CIBERCV-ISCIII, A Coruña, Spain; [e]Cardiovascular Division, Brigham and Women's Hospital, Harvard Medical School, Boston, MA, USA; [f]School of Cardiovascular and Metabolic Health, University of Glasgow, Glasgow, Scotland; [g]Brigham and Women's Hospital, Boston, MA, USA; [h]Division of Cardiology, Department of Medicine, Massachusetts General Hospital, Harvard Medical School, Boston, MA, USA; [i]Baim Institute for Clinical Research, Boston, MA, USA; [j]Oregon Health & Science University, Portland, OR, USA; [k]University of Missouri Kansas City Healthcare Institute for Innovations in Quality and Saint Luke's Mid America Heart Institute, Kansas City, MO, USA; [l]Saint Luke's Mid America Heart Institute, Kansas City, MO, USA; [m]Cytokinetics, Incorporated, South San Francisco, CA, USA; [n]Meyer Children's Hospital, Instituto di Ricovero e Cura a Carattere Scientifico (IRCCS), Florence, Italy

Table of Contents

Supplementary Table 1 2

Supplementary Table 2 3

Supplementary Table 3 4

Supplementary Table 4 6

Supplementary Table 1. Sensitivity analysis comparing efficacy and safety outcomes in symptomatic obstructive HCM patients on aficamten and patients excluded at baseline considered in the mild symptom group

	Symptom Groups on Aficamten Treatment*		
	Mild Symptoms† n=75	Moderate to Severe Symptoms‡ n=71	
**Efficacy Outcomes**	**Treatment Effect, Mean (95% CI)**	**Treatment Effect, Mean (95% CI)**	**Interaction *P*-value**
**pVO_2, Week 24, mL/kg/min**	1.6 (0.6, 2.6)	1.8 (0.8, 2.8)	0.76
**KCCQ-CSS at Week 24**	4 (2, 6)	10 (6, 15)	0.02
**≥1 NYHA FC improvement, Week 24, %**	35 (19, 51)	34 (19, 49)	0.92
**Valsalva LVOT-G, Week 24, mmHg**	−53 (−62, −44)	−47 (−57, −37)	0.36
**Resting LVOT-G, Week 24, mmHg**	−41 (−49, −33)	−38 (−47, −29)	0.65
**Valsalva LVOT-G <30 mmHg, Week 24, %**	54 (41, 67)	37 (25, 50)	0.06
**NT-proBNP change, Week 24, %**	−80 (−84, −75)	−81 (−85, −76)	0.90
**LA volume index, Week 24, mL/m^2**	−4.5 (−7.1, −1.9)	−3.3 (−5.5 −1.2)	0.48
**Lateral E/e′**	−4.9 (−6.8, −2.9)	−3.1 (−4.2, −1.9)	0.10
**Sepal E/e′**	−4.1 (−5.7, −2.5)	−3.4 (−5.1, −1.7)	0.60
**Maximum LV wall thickness, cm**	−0.15 (−0.24, −0.06)	−0.11 (−0.18, −0.03)	0.49
**Safety outcomes, aficamten vs. placebo, n (%)**	**Mild Symptoms† n=132**	**Moderate to Severe Symptoms‡ n=150**	
**Any adverse event (TE)**	54 (76) vs. 38 (62)	51 (72) vs. 61 (77)	
**Any serious adverse event (TE)**	0 (0) vs. 5 (8)	8 (11) vs. 8 (10)	
**LVEF <50%**	3 (4) vs. 0 (0)	2 (3) vs. 1 (1)	

*Includes in the mild symptoms group the 14 patients assigned to NYHA functional class III but reported low symptom burden (KCCQ-CSS ≥80) at BL and were excluded from the primary analysis.
†Defined as patients with NYHA functional class II and KCCQ-CSS ≥80.
‡Defined as patients with NYHA functional class II/III/IV and KCCQ-CSS <80.
BL, baseline; CI, confidence interval; E/e¢, peak E wave to annual diastolic velocity ratio; HCM, hypertrophic cardiomyopathy; KCCQ-CSS, Kansas City Cardiomyopathy Questionnaire-Clinical Summary Score; LA, left atrial; LV, left ventricular; LVEF, left ventricular ejection fraction; LVOT-G, left ventricular outflow tract gradient; NT-proBNP, N-terminal pro–B-type natriuretic peptide; NYHA, New York Heart Association functional class; pVO_2, peak oxygen uptake; TE, treatment-emergent.

Supplementary Table 2. Sensitivity analysis comparing efficacy and safety outcomes in symptomatic obstructive HCM patients on aficamten and patients excluded at baseline considered in the moderate to severe symptom group

	**Symptom Groups on Aficamten Treatment***		
	**Mild Symptoms†**	**Moderate to Severe Symptoms‡**	
**Efficacy Outcomes**	**Treatment Effect, Mean (95% CI)**	**Treatment Effect, Mean (95% CI)**	**Interaction *P*-value**
**pVO_2**, Wee**k 24, mL/kg/min**	1.6 (0.5, 2.7)	1.8 (0.8, 2.7)	0.89
**KCCQ-CSS**, Wee**k 24**	4 (1, 6)	10 (6, 14)	0.03
**≥1 NYHA** FC **improvement**, Week **24, %**	33 (16, 49)	36 (22, 51)	0.73
**Valsalva LVOT**-G, Wee**k 24, mmHg**	−53 (−62, −44)	−48 (−58, −38)	0.53
**Resting LVOT**-G, Wee**k 24, mmHg**	−41 (−49, −33)	−38 (−47, −30)	0.64
**Valsalva LVOT**-G **<30 mmHg**, Wee**k 24, %**	54 (40, 68)	39 (28, 51)	0.10
**NT-proBNP change**, Wee**k 24, %**	−79 (−83, −73)	−82 (−85, −77)	0.32
**LA volume index**, Wee**k 24, mL/m²**	−4.6 (−7.3, −1.9)	−3.3 (−5.5 −1.2)	0.45
**Lateral E/e′**	−4.3 (−6.4, −2.3)	−3.6 (−4.8, −2.4)	0.38
**Sepal E/e′**	−3.4 (−5.1, −1.8)	−3.9 (−5.5, −2.3)	0.76
**Max**imum **LV wall thickness**	−0.16 (−0.25, −0.07)	−0.10 (−0.18, −0.02)	0.33

**Safety outcomes, aficamten vs. placebo, n (%)**	**Mild Symptoms† n=118**	**Moderate to Severe Symptoms‡ n=164**	
**Any adverse event (TE)**	49 (79) vs. 35 (63)	56 (70) vs. 64 (76)	–
**Any serious adverse event (TE)**	0 (0) vs. 5 (9)	8 (10) vs. 8 (10)	–
**LVEF <50%**	3 (5) vs. 0 (0)	2 (3) vs. 1 (1)	–

*Includes in the moderate to severe symptoms group the 14 patients assigned to NYHA functional class III but reported low symptom burden (KCCQ-CSS ≥80) at BL and excluded from the primary analysis.
†Defined as patients with NYHA FC II and KCCQ-CSS ≥80.
‡Defined as patients with NYHA FC II/III/IV and KCCQ-CSS <80.
BL, baseline; CI, confidence interval; E/e¢, peak E wave to annual diastolic velocity ratio; HCM, hypertrophic cardiomyopathy; KCCQ-CSS, Kansas City Cardiomyopathy Questionnaire-Clinical Summary Score; LA, left atrial; LV, left ventricular; LVEF, left ventricular ejection fraction; LVOT-G, left ventricular outflow tract gradient; NT-proBNP, N-terminal pro–B-type natriuretic peptide; NYHA FC, New York Heart Association functional class; pVO_2, peak oxygen uptake; TE, treatment-emergent.

Supplementary Table 3. Demographic and clinical characteristics of patients with obstructive HCM and mild symptoms assigned to aficamten vs placebo*

	**Mild Symptoms on Placebo n=56**	**Mild Symptoms on Aficamten n=62**	***P*-value**
Randomized to active treatment	0 (0.0)	62 (100.0)	<0.001
Age, y	57 ± 14	59 ± 13	0.43
Female sex	18 (32.1)	18 (29.0)	0.71
Race			0.21
Asian	16 (28.6)	21 (33.9)	
Black or African American	0 (0.0)	2 (3.2)	
Other	0 (0.0)	2 (3.2)	
White	40 (71.4)	37 (59.7)	
Geographic region			0.75
China	14 (25.0)	17 (27.4)	
North America	12 (21.4)	16 (25.8)	
Rest of World	30 (53.6)	29 (46.8)	
Medical history			
Hypertension	24 (42.9)	33 (53.2)	0.26
Known HCM-causing gene mutation	8 (14.3)	7 (11.3)	0.63
Positive family history of HCM	10 (17.9)	18 (29.0)	0.15
Paroxysmal atrial fibrillation	8 (14.3)	6 (9.7)	0.44
Coronary artery disease	5 (8.9)	10 (16.1)	0.24
Diabetes	4 (7.1)	8 (12.9)	0.30
Permanent atrial fibrillation	0 (0.0)	2 (3.2)	0.18
Vital signs			
Systolic blood pressure at BL, mmHg	125 ± 15	124 ± 14	0.66
Diastolic blood pressure at BL, mmHg	76 ± 10	74 ± 11	0.57
Resting heart rate, bpm	70 ± 12	67 ± 11	0.12
BMI at BL, kg/m^2	28 ± 3	27 ± 4	0.18
Background HCM therapy			
Beta-blocker	33 (58.9)	34 (54.8)	0.65
Calcium channel blocker	15 (26.8)	26 (41.9)	0.08
Selective calcium channel blockers with direct cardiac effects	10 (17.9)	22 (35.5)	0.032
Selective calcium channel blockers with mainly vascular effects	5 (8.9)	5 (8.1)	0.87

Disopyramide use at BL	9 (16.1)	4 (6.5)	0.10
History: ICD insertion	7 (12.5)	8 (12.9)	0.95
KCCQ-CSS at BL	89 ± 5	90 ± 6	0.21
NYHA functional class at BL	56 (100.0)	62 (100.0)	–
NT-proBNP at BL, pg/mL	797 [280, 1766]	830 [370, 1544]	0.79
hsTroponin I type 3 at BL, ng/L	13 [8, 40]	16 [11, 38]	0.50
CPET			
CPET modality: Bicycle	26 (46.4)	32 (51.6)	0.57
CPET modality: Treadmill	30 (53.6)	30 (48.4)	0.57
Total workload CPET at BL, watts	130 ± 38	124 ± 40	0.44
pVO_2 CPET at BL, mL/kg/min	20 ± 4	19 ± 5	0.41
% Predicted oxygen uptake	60 ± 14	57 ± 11	0.22
Peak respiratory exchange ratio	1 ± 0	1 ± 0	0.10
Echocardiographic parameters			
Core labs peak Valsalva LVOT-G, mmHg	81.2 ± 29.4	81.5 ± 37.2	0.97
Core labs LVEF at BL, %	74.8 ± 6.8	75.2 ± 5.2	0.73
Core labs peak resting LVOT-G, mmHg	54.3 ± 28.1	53.7 ± 29.9	0.91
LV maximal wall thickness, cm	2.2 ± 0.3	2.1 ± 0.3	0.09
BSA indexed LAV at BL, mL/m^2	40.6 ± 13.9	38.9 ± 10.1	0.44
Septal E/e′	20.3 ± 10.7	18.8 ± 7.6	0.40
Lateral E/e′	16.0 ± 8.8	15.0 ± 6.9	0.50

Data are n (%), mean ± SD, or median [range].
*Mild symptoms was defined as NYHA class II and KCCQ-CSS ≥80.
BL, baseline; BMI, body mass index; BSA, body surface area; CPET, cardiopulmonary exercise test; E/e¢, peak E wave to annular early diastolic velocity ratio; HCM, hypertrophic cardiomyopathy; HS, high sensitivity; ICD, Implantable cardioverter defibrillator; KCCQ-CSS, Kansas City Cardiomyopathy Questionnaire-Clinical Summary Score; LAV, left atrial volume; LV, left ventricular; LVEF, left ventricular ejection fraction; LVOT-G, left ventricular outflow tract gradient; NT-proBNP, N-terminal pro-B-type natriuretic peptide; NYHA, New York Heart Association; pVO_2, peak oxygen uptake.

Supplementary Table 4. Demographic and clinical characteristics of patients with obstructive HCM and moderate to severe symptoms assigned to aficamten vs placebo*

	**Moderate to Severe Symptoms on Placebo n=79**	**Moderate to Severe Symptoms on Aficamten n=71**	***P*-value**
Randomized to active treatment	0 (0.0)	71 (100.0)	p<0.001
Age, y	60 ± 13	59 ± 12	0.53
Female sex	39 (49.4)	33 (46.5)	0.72
Race			0.49
Asian	9 (11.4)	6 (8.5)	
Black or African American	0 (0.0)	1 (1.4)	
White	70 (88.6)	64 (90.1)	
Geographic Region			0.80
China	8 (10.1)	5 (7.0)	
North America	30 (38.0)	28 (39.4)	
Rest of World	41 (51.9)	38 (53.5)	
Medical History			
Hypertension	44 (55.7)	39 (54.9)	0.92
Known HCM-causing gene mutation	15 (19.0)	15 (21.1)	0.74
Positive family history of HCM	23 (29.1)	21 (29.6)	0.95
Paroxysmal atrial fibrillation	11 (13.9)	14 (19.7)	0.34
Coronary artery disease	9 (11.4)	7 (9.9)	0.76
Diabetes	5 (6.3)	6 (8.5)	0.62
Permanent atrial fibrillation	1 (1.3)	0 (0.0)	0.34
Vital signs			
Systolic blood pressure at BL, mmHg	125 ± 17	125 ± 17	0.86
Diastolic blood pressure at BL, mmHg	73 ± 11	75 ± 11	0.33
Resting heart rate, bpm	71 ± 14	70 ± 11	0.65
BMI at BL, kg/m^2	28 ± 4	29 ± 4	0.10
Background HCM therapy			
Beta-blocker	50 (63.3)	44 (62.0)	0.87
Calcium channel blocker	30 (38.0)	25 (35.2)	0.73
Selective calcium channel blockers with direct cardiac effects	25 (31.6)	23 (32.4)	0.92
Selective calcium channel blockers with mainly vascular effects	5 (6.3)	2 (2.8)	0.31

Disopyramide use at BL	10 (12.7)	11 (15.5)	0.62
History: ICD insertion	9 (11.4)	12 (16.9)	0.33
KCCQ-CSS at BL	62 ± 14	61 ± 15	0.90
NYHA functional class at BL			0.63
Class II	50 (63.3)	46 (64.8)	
Class III	28 (35.4)	25 (35.2)	
Class IV	1 (1.3)	0 (0.0)	
NT-proBNP at BL, pg/mL	644 [344, 1850]	746 [346, 1630]	0.88
hsTroponin I type 3 at BL, ng/L	10 [7, 20]	10 [7, 27]	0.88
CPET			
CPET Modality: Bicycle	34 (43.0)	28 (39.4)	0.65
CPET Modality: Treadmill	45 (57.0)	43 (60.6)	0.65
Total workload CPET at BL, watts	120 ± 43	118 ± 38	0.80
pVO_2 CPET at BL, mL/kg/min	18 ± 5	18 ± 4	0.84
% Predicted oxygen uptake	57 ± 12	55 ± 10	0.39
Peak respiratory exchange ratio	1 ± 0	1 ± 0	0.74
Echocardiographic parameters			
Core labs peak Valsalva LVOT-G, mmHg	85.8 ± 35.1	82.7 ± 26.9	0.56
Core labs LVEF at BL, %	74.8 ± 6.0	74.6 ± 5.9	0.81
Core labs peak resting LVOT-G, mmHg	56.6 ± 35.5	55.0 ± 24.2	0.75
LV maximal wall thickness, cm	2.1 ± 0.3	2.1 ± 0.3	0.89
BSA indexed LAV at BL, mL/m^2	41.1 ± 16.2	40.2 ± 14.4	0.71
Septal E/e′	20.6 ± 8.3	19.5 ± 7.9	0.40
Lateral E/e′	15.8 ± 7.1	15.4 ± 7.4	0.72

Data are n (%), mean ± SD, or median [range].
*Moderate to severe symptoms was defined as NYHA class II/III/IV and KCCQ-CSS <80.
BL, baseline; BMI, body mass index; BSA, body surface area; CPET, cardiopulmonary exercise test; E/e¢, peak E wave to annular early diastolic velocity ratio; HCM, hypertrophic cardiomyopathy; HS, high sensitivity; ICD, Implantable cardioverter defibrillator; KCCQ-CSS, Kansas City Cardiomyopathy Questionnaire-Clinical Summary Score; LAV, left atrial volume; LV, left ventricular; LVEF, left ventricular ejection fraction; LVOT-G, left ventricular outflow tract gradient; NT-proBNP, N-terminal pro-B-type natriuretic peptide; NYHA, New York Heart Association; pVO_2, peak oxygen uptake.
